# Interleukin‐17A, Interleukin‐8, and Platelet Count Are Associated With Baseline Coronary Artery Lesions in Children With Kawasaki Disease: A Retrospective Exploratory Cohort Study

**DOI:** 10.1002/iid3.70514

**Published:** 2026-09-24

**Authors:** Cuijie Gong, Yahui Lin, Yanmei Lang, Xinfeng Liu, Dandan Li, Sha Wang, Pan Li

**Affiliations:** ^1^ Department of Respiratory Medicine V (International Department) Hebei Children's Hospital, Hebei Clinical Research Center for Children's Health and Diseases Shijiazhuang China

**Keywords:** baseline coronary involvement, coronary artery lesions, IL‐17A, IL‐8, internal validation, Kawasaki disease, platelets, Z‐scores

## Abstract

**Background:**

Kawasaki disease (KD) is an acute childhood vasculitis in which coronary artery lesions (CALs) determine long‐term cardiovascular risk. We evaluated a focused pre‐IVIG biomarker profile in relation to baseline Z‐score‐defined coronary involvement.

**Objective:**

To examine whether interleukin‐17A (IL‐17A), interleukin‐8 (IL‐8), and platelet count (PLT) were associated with baseline CAL and to assess the robustness and internal discrimination of this exploratory profile.

**Methods:**

This single‐center retrospective exploratory cohort study screened 242 children with suspected KD treated in 2023. After 43 exclusions, 199 children were analyzed (non‐CAL, *n* = 166; CAL, *n* = 33). CAL was defined using pretreatment segment‐level coronary Z‐scores recorded in the institutional echocardiographic database. To limit model complexity, the primary Firth‐penalized logistic model was restricted to three predictors (PLT, IL‐8, and IL‐17A; 11 events per predictor). A nine‐covariate clinically informed model and illness‐timing/incomplete‐KD models were treated as sensitivity analyses. Internal validation used 1000‐resample bootstrap optimism correction and 100 repeated stratified fivefold cross‐validation with complete refitting.

**Results:**

Compared with the non‐CAL group, children with CAL had higher PLT, IL‐6, IL‐8, and IL‐17A and lower hematocrit (HCT), among other group‐level differences. In the primary Firth model, PLT (OR, 3.081 per 100 × 10^9^/L; 95% CI, 1.582–6.002), IL‐8 (OR, 1.167 per 10 pg/mL; 95% CI, 1.076–1.266), and IL‐17A (OR, 6.555 per 10 pg/mL; 95% CI, 3.054–14.071) were associated with baseline CAL. The apparent AUC was 0.975, the optimism‐corrected AUC was 0.972, and the repeated‐cross‐validation mean AUC was 0.966 (SD, 0.006).

**Conclusions:**

Higher pre‐IVIG IL‐17A, IL‐8, and PLT were associated with baseline Z‐score‐defined CAL. These internally validated but single‐center observational findings neither establish mechanism nor justify biomarker‐guided imaging or treatment. Prospective multicenter external validation is required before clinical use.

## Graphical abstract

Early risk stratification for coronary artery lesions in Kawasaki disease

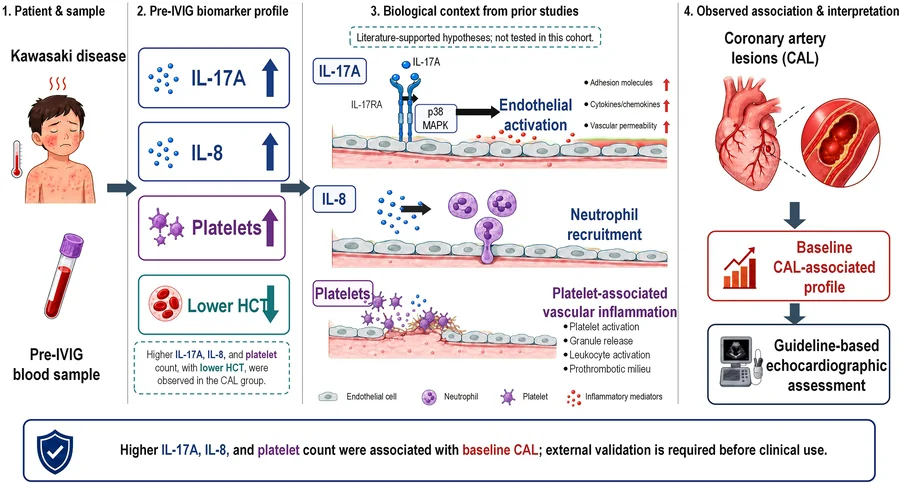

**Abbreviations**
Abbreviation: definition
AHA: American Heart Association
AUC: area under the receiver operating characteristic curve
BSA: body surface area
CAL: coronary artery lesion
CI: confidence interval
CRP: C‐reactive protein
ESR: erythrocyte sedimentation rate
Hb: hemoglobin
HCT: hematocrit
IL: interleukin
IVIG: intravenous immunoglobulin
JCS: Japanese Circulation Society
JSCS: Japanese Society for Cardiovascular Surgery
KD: Kawasaki disease
OR: odds ratio
PLT: platelet count
ROC: receiver operating characteristic
SD: standard deviation
STROBE: Strengthening the Reporting of Observational Studies in Epidemiology
Th17: T helper 17
WBC: white blood cell

## 1 Introduction

Kawasaki disease (KD) is an acute, self‐limited, medium‐vessel vasculitis that predominantly affects infants and young children, but its consequences can persist across the life course when the coronary arteries are involved [1, 2, 3]. The clinical urgency of KD derives from a distinctive combination of diagnostic uncertainty and preventable cardiovascular injury. Timely administration of intravenous immunoglobulin (IVIG) markedly reduces the frequency of coronary artery aneurysms, yet coronary dilation, aneurysmal change, thrombosis, stenosis, and late ischemic events still occur in a clinically important minority of patients [1, 2, 4]. Contemporary management of KD is therefore not limited to recognizing the acute clinical syndrome; it also requires careful assessment of coronary involvement and inflammatory phenotype in children who may require intensified echocardiographic surveillance, early cardiology involvement, or adjunctive anti‐inflammatory therapy [1, 2, 3].

Conventional markers such as CRP, ESR, and IL‐6 reflect systemic inflammation rather than coronary‐specific vascular injury. Whether a more focused cytokine‐centered profile is associated with baseline coronary involvement remains uncertain [1, 2, 5].

Epidemiologic evidence underscores the continuing relevance of assessing coronary involvement and coronary‐risk‐related inflammatory phenotypes in KD. KD incidence is highest in East Asian populations, but the disease occurs worldwide and remains the leading cause of acquired heart disease in children in many high‐income settings [6, 7, 8]. Large surveys from China and Japan show that coronary involvement still occurs despite improved diagnostic awareness and widespread IVIG use [7, 8]. These data also suggest that demographic factors, timing of treatment, platelet indices, albumin, inflammatory burden, and IVIG resistance contribute to coronary outcomes [7, 8]. Current American Heart Association (AHA) guidance and Japanese cardiovascular sequelae guidelines emphasize body‐surface‐area‐adjusted coronary artery z‐scores for coronary classification and longitudinal risk management, because absolute coronary diameters can misclassify young children with different body sizes [1, 2, 9, 10]. Thus, laboratory markers should not be described as diagnostic substitutes for echocardiography. Their appropriate role is to contextualize inflammatory status alongside echocardiographic assessment rather than replace standardized coronary imaging.

Several clinical risk scores have been developed in KD, particularly to identify children at risk of IVIG nonresponse. The Kobayashi, Egami, and Sano scores were derived in Japanese populations and have been widely used to guide risk‐adapted initial therapy [11, 12, 13]. However, external validation studies have shown variable sensitivity and calibration outside Japan, and baseline coronary z‐scores may outperform laboratory‐only scores in some North American cohorts [14, 15, 16]. More recent CAL‐specific nomograms, biomarker panels, and machine‐learning approaches have incorporated inflammatory markers, hemoglobin, albumin, CRP, neutrophil‐to‐lymphocyte ratio, lipid variables, and computational algorithms [17, 18, 19, 20, 21, 22]. These studies confirm the clinical demand for better coronary assessment, but they also reveal a recurring limitation: many candidate variables are easy to measure but biologically nonspecific.

Cytokines may help contextualize coronary inflammation, but observational biomarker data cannot by themselves establish a causal pathway. Early work demonstrated dynamic elevations of IL‐6, IL‐8, and tumor necrosis factor‐α during acute KD and suggested associations with coronary aneurysm formation [23]. IL‐8 promotes neutrophil chemotaxis, adhesion, and transmigration [24]. IL‐6 is associated with KD activity and shock phenotype but often behaves as a broad acute‐phase signal [25, 26]. Recent studies have focused on the Th17/IL‐17 axis, particularly IL‐17A [27, 28, 29].

Prior proteomic and translational studies have already linked IL‐17‐family signals, IL‐8 expression, neutrophil recruitment, and coronary arteritis in KD [27, 28, 29, 30, 31, 32]. The present study does not introduce a new cytokine or experimentally test these mechanisms. Its narrower contribution is a simultaneous pre‐IVIG evaluation of IL‐17A, IL‐8, and PLT against recorded segment‐level coronary Z‐scores, with explicit small‐sample correction and internal validation. Any mechanistic interpretation is therefore literature‐supported and hypothesis‐generating rather than demonstrated in this cohort.

A recent clinical report likewise described elevated IL‐17A at KD onset and emphasized the need for larger validation studies [33]. Replication across independent clinical datasets is useful, but it should not be mistaken for mechanistic novelty.

This retrospective cohort study assessed whether a focused three‐marker profile comprising IL‐17A, IL‐8, and PLT was associated with baseline CAL. We treated the analysis as exploratory association research, not as prediction of incident CAL, a deployable clinical rule, or a mechanistic experiment.

## 2 Methods

### 2.1 Study Design and Reporting Framework

This single‐center retrospective exploratory cohort study was conducted at Hebei Children's Hospital and included children with KD treated between January 2023 and December 2023. Reporting followed the Strengthening the Reporting of Observational Studies in Epidemiology (STROBE) recommendations [34]. The outcome was CAL already present on the pretreatment echocardiogram; therefore, the analyses concern cross‐sectional associations within a retrospective cohort and not future CAL prediction. No validated prediction model or deployable clinical rule was developed [35, 36].

### 2.2 Participants and Eligibility Criteria

**Figure 1:**
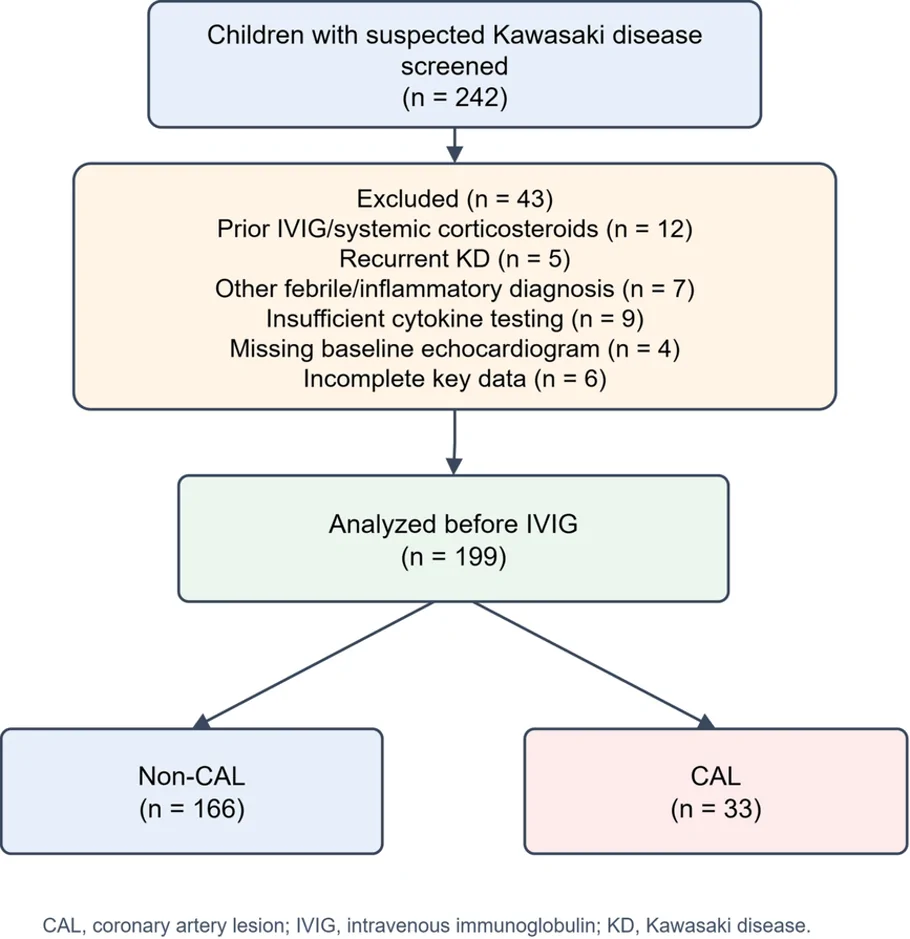
Participant flow. A total of 242 children with suspected Kawasaki disease were screened; 43 were excluded for the stated reasons and 199 were analyzed (non‐CAL, *n* = 166; CAL, *n* = 33). CAL, coronary artery lesion; IVIG, intravenous immunoglobulin.

Eligible patients were children with a first episode of KD who met AHA diagnostic criteria and who had clinical, laboratory, cytokine, and echocardiographic data available before IVIG administration [1, 2]. Patients were excluded if they had received IVIG or systemic corticosteroids before admission, had recurrent KD, had incomplete records preventing group classification, had another identified febrile or inflammatory disease explaining the presentation, or lacked key laboratory or echocardiographic data. The screening log was reviewed to characterize patient selection. A total of 242 children with suspected KD were initially screened. Forty‐three patients were excluded for specific reasons: received IVIG or systemic corticosteroids before admission (*n* = 12), recurrent KD (*n* = 5), final diagnosis of other febrile/inflammatory disease (*n* = 7), missing or insufficient cytokine testing (*n* = 9), missing baseline echocardiogram (*n* = 4), and incomplete key clinical/laboratory data (*n* = 6). The final analytic cohort comprised 199 children. Age and sex were compared between included and excluded patients; other clinical and inflammatory characteristics were not available for all excluded patients. According to the presence or absence of echocardiographically documented coronary involvement, 166 children were assigned to the non‐CAL group and 33 children to the CAL group (Figure 1). The study protocol was approved by the Ethics Committee of Hebei Children's Hospital (approval No. 202136), and written informed consent for clinical data use was obtained from legal guardians according to local requirements.

### 2.3 Coronary Artery Assessment

Transthoracic echocardiography was performed before IVIG treatment. After re‐review of the archived institutional echocardiographic database, the absolute internal diameters and corresponding segment‐level Z‐scores recorded at the time of care were recovered for the left main coronary artery (LMCA), left anterior descending artery (LAD), right coronary artery (RCA), and left circumflex artery (LCX). These Z‐scores were retrieved as archived clinical values and were not recalculated from the reconstructed body surface area (BSA) data. BSA was calculated from admission height and weight using the Mosteller formula for descriptive reporting only. CAL was defined as a maximum recorded segmental Z‐score ≥ 2.0. Severity categories were dilation only (Z 2.0 to < 2.5), small aneurysm (Z 2.5 to < 5.0), medium aneurysm (Z 5.0 to < 10.0 and absolute dimension < 8 mm), and large/giant aneurysm (Z ≥ 10.0 or absolute dimension ≥ 8 mm) [1, 2, 9, 10]. Two echocardiographers interpreted examinations, with discrepancies resolved by a third senior echocardiographer. The archived file did not retain the Z‐score equation or software version, which limits method reproducibility and cross‐study comparability. Individual CAL‐case measurements are provided in Supplementary Table S1.

### 2.4 Laboratory and Cytokine Measurements

Peripheral venous blood samples were collected during the acute phase before IVIG treatment, generally on the day of admission and before immunomodulatory therapy. The blood‐sampling illness day therefore corresponded to the admission illness day and was contemporaneous with baseline echocardiography. Routine variables included WBC count, neutrophil percentage, Hb, HCT, PLT, CRP, ESR, fibrinogen, D‐dimer, albumin, prealbumin, ALT, AST, sodium, ferritin, and BNP. The complete measured cytokine panel comprised IL‐2, IL‐4, IL‐6, IL‐8, IL‐10, TNF‐α, IFN‐γ, IL‐17A, IL‐1β, IL‐5, IL‐12p70, and IFN‐α. Cytokines were quantified using the ABplex Human Cytokine 12‐Plex Assay Kit (catalog no. RK04296A; ABclonal Technology Co. Ltd., Wuhan, China), a magnetic‐bead multiplex immunoassay, according to the manufacturer's instructions. Samples were analyzed on an ABplex‐100 Analyzer (ABclonal Technology Co. Ltd., Wuhan, China), and concentrations were reported in pg/mL. The manufacturer‐reported lower limits of detection were 2.5 pg/mL for IL‐6, IL‐8, and IL‐17A. The laboratory reference intervals were 0–5.30 pg/mL for IL‐6, 0–20.6 pg/mL for IL‐8, and 0–20.60 pg/mL for IL‐17A.

### 2.5 Statistical Analysis

Continuous variables were summarized as mean ± SD or median (IQR), as appropriate; categorical variables were summarized as *n* (%). Group comparisons used t/Welch, rank‐based, or chi‐square tests. All values in tables and figures follow consistent rounding: continuous summaries generally use two decimals, ORs/AUCs use three decimals, percentages use one decimal, and *p* values use three decimals or < 0.001. Apparent discrimination was summarized using the area under the receiver operating characteristic curve (AUC), and pairwise comparisons of correlated single‐marker AUCs used the DeLong method [37, 38]. Because only 33 CAL events were available, the primary association model was restricted to PLT, IL‐8, and IL‐17A (three predictors; 11 events per predictor) to reduce model complexity; this reduced model should be regarded as exploratory rather than prospectively specified [39]. Firth bias‐reduced logistic regression was used as the primary estimator to reduce small‐sample and separation bias [40]. PLT was expressed per 100 × 10^9^/L and cytokines per 10 pg/mL. A conventional maximum‐likelihood version was reported as a sensitivity analysis.

### 2.6 Robustness and Discrimination Analyses

A nine‐covariate clinically informed Firth model (age, sex, illness day, albumin, sodium, HCT, PLT, IL‐8, and IL‐17A) was retained as an explicitly exploratory expanded analysis. Additional Firth models added either admission illness day plus incomplete‐KD status or fever duration before IVIG plus incomplete‐KD status to the three primary biomarkers. IVIG resistance was not adjusted for because it was determined after the baseline echocardiographic outcome; time from admission to IVIG was likewise post‐outcome and was summarized descriptively. IL‐6 was retained in the univariate, complete‐cytokine‐panel, and single‐marker discrimination analyses but was not added to the reduced primary multivariable model because doing so would increase predictor burden in a cohort with only 33 CAL events. Apparent AUCs were reported without data‐derived thresholds. The primary model underwent 1000‐resample bootstrap optimism correction and 100 repetitions of stratified fivefold cross‐validation with complete refitting. Two‐sided *p* < 0.05 was considered statistically significant. Analyses used SPSS 27.0 and R 4.6.1 (logistf and pROC packages).

## 3 Results

### 3.1 Baseline Characteristics and Patient Selection

Of 242 children screened, 43 were excluded; 199 were analyzed (non‐CAL, *n* = 166; CAL, *n* = 33). Included and excluded children were similar in age and sex, although other variables were not consistently available for excluded patients. Table 1 now reports sex, age, admission/blood‐sampling illness day, fever duration before IVIG, time from admission to IVIG, number of principal KD features, complete versus incomplete KD, and IVIG resistance. Children with CAL presented and received IVIG later and had more IVIG resistance. Because CAL was already present on baseline echocardiography, IVIG resistance and admission‐to‐IVIG time were treated as descriptive post‐outcome variables rather than confounders in the primary association model.

**Table 1:** Demographic, disease‐timing, and treatment‐response characteristics.

Characteristic	Non‐CAL (*n* = 166)	CAL (*n* = 33)	Test statistic	*p* value
Male sex, *n* (%)	106 (63.9)	24 (72.7)	χ^2^ = 0.96	0.328
Age, years	2.78 ± 2.08	2.34 ± 2.16	t = 1.06	0.292
Admission/blood‐sampling illness day, days	6.0 (5.0–7.0)	7.0 (6.0–8.0)	Z = 2.54	0.011
Fever duration before IVIG, days	6.0 (5.0–8.0)	7.0 (6.0–9.0)	Z = 2.43	0.015
Time from admission to IVIG, hours	10.30 ± 3.65	13.27 ± 4.23	t = 3.86	< 0.001
Principal KD features fulfilled, *n*	4.0 (4.0–4.75)	4.0 (3.0–5.0)	Z = 0.83	0.404
Complete KD, *n* (%)	126 (75.9)	20 (60.6)	χ^2^ = 3.31	0.069
Incomplete KD, *n* (%)	40 (24.1)	13 (39.4)	χ^2^ = 3.31	0.069
IVIG resistance, *n* (%)	13 (7.8)	9 (27.3)	χ^2^ = 10.66	0.001

*Note:* Data are mean ± SD, median (IQR), or *n* (%). CAL was present on baseline echocardiography. IVIG resistance and admission‐to‐IVIG time are post‐outcome characteristics and are descriptive, not primary‐model covariates.

Abbreviations: CAL, coronary artery lesion; IVIG, intravenous immunoglobulin; KD, Kawasaki disease.

### 3.2 Univariate Biomarker Differences

**Figure 2:**
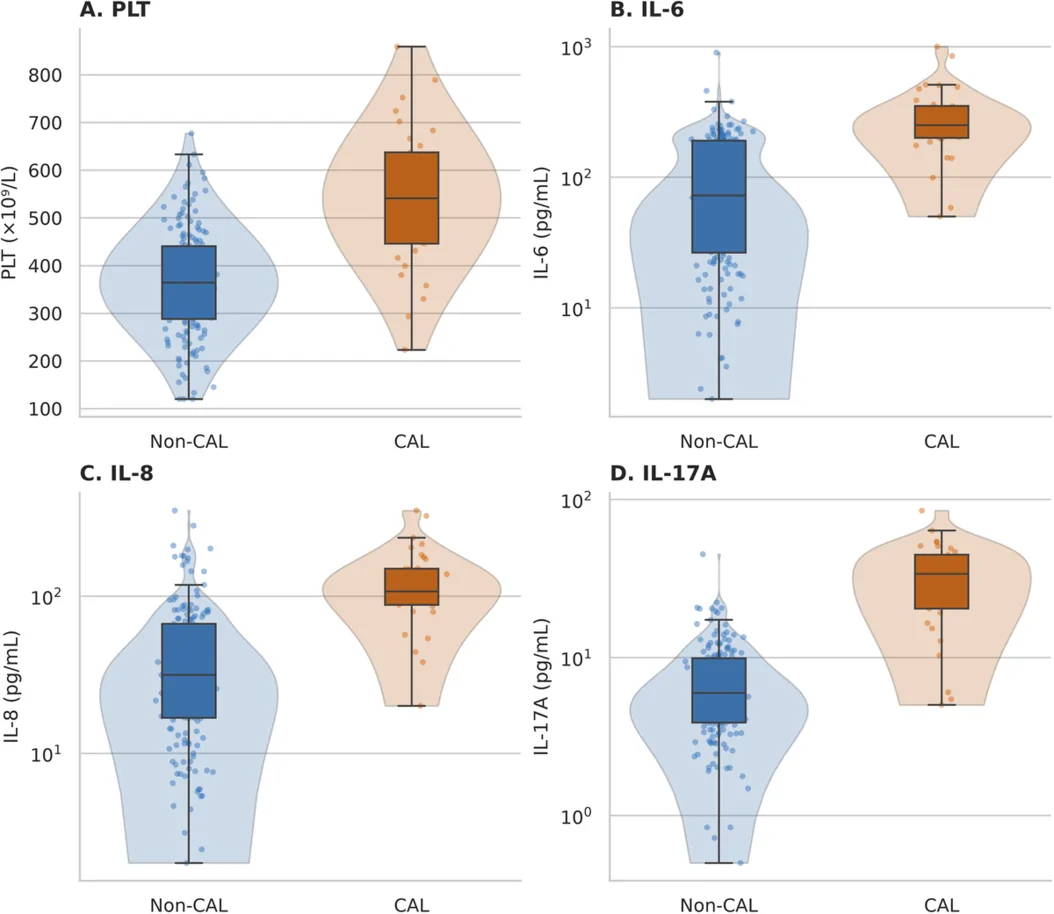
Individual distributions of PLT, IL‐6, IL‐8, and IL‐17A among children without CAL (*n* = 166) and with CAL (*n* = 33). Violin density, box plots, and jittered patient‐level observations are shown. Cytokine axes are logarithmic because of right‐skewed distributions. Boxes show the interquartile range and median. CAL, coronary artery lesion; IL, interleukin; PLT, platelet count.

Univariate comparisons showed a consistent pattern of greater inflammatory and hematologic perturbation in the CAL group (Table 2). PLT was substantially higher in children with CALs than in those without CALs (541.06 ± 146.24 vs. 364.81 ± 112.29 × 10^9^/L, *p* < 0.001). ESR was also higher in the CAL group (89.76 ± 21.55 vs. 64.90 ± 23.86 mm/h, *p* < 0.001), whereas HCT was lower (0.31 ± 0.04 vs. 0.34 ± 0.03, *p* < 0.001). Skewed variables showed similar separation: WBC count, CRP, d‐dimer, ALT, AST, BNP, ferritin, IL‐6, IL‐8, and IL‐17A were all higher in the CAL group, while Hb, albumin, prealbumin, and sodium were lower. IL‐17A displayed the largest separation among measured cytokines, with a median of 33.80 (interquartile range, 20.40–44.60) in the CAL group compared with 5.96 (3.87–9.87) in the non‐CAL group (*p* < 0.001). The direction and relative magnitude of these group‐level contrasts for the representative primary markers are displayed in Figure 2. Results for the complete 12‐cytokine panel are provided in Table S2.

### 3.3 Echocardiographic Coronary Outcomes

Segment‐level coronary measurements are summarized in Table 3. LMCA, LAD, and RCA internal diameters were larger in the CAL group, whereas the difference in LCX diameter was not statistically significant. LMCA, LAD, RCA, LCX, and maximum Z‐scores were higher in the CAL group. The median maximum Z‐score was 2.62 (IQR, 2.23–3.81) in the CAL group versus 1.04 (IQR, 0.63–1.30) in the non‐CAL group (*p* < 0.001). Among the 33 children with CAL, 15 (45.5%) had dilation only, 12 (36.4%) had small aneurysms, 5 (15.2%) had medium aneurysms, and 1 (3.0%) had a large/giant aneurysm. Twenty‐four children had single‐segment involvement and nine had two‐segment involvement.

**Table 2:** Laboratory and cytokine comparisons.

Variable	Non‐CAL (*n* = 166)	CAL (*n* = 33)	*p* value
WBC, × 10^9^/L	14.70 (12.20–18.60)	17.90 (15.80–19.80)	0.002
Neutrophils, %	70.50 ± 12.00	76.00 ± 12.00	0.020
Hb, g/L	111.00 (105.00–118.00)	105.00 (97.00–116.00)	0.011
HCT	0.34 ± 0.03	0.31 ± 0.04	< 0.001
PLT, × 10^9^/L	364.81 ± 112.29	541.06 ± 146.24	< 0.001
CRP, mg/L	60.73 (36.74–91.65)	90.77 (76.25–122.90)	< 0.001
ESR, mm/h	64.90 ± 23.86	89.76 ± 21.55	< 0.001
Fibrinogen, g/L	5.30 ± 1.50	6.20 ± 1.50	0.003
D‐dimer, mg/L FEU	0.65 (0.35–1.06)	1.05 (0.65–1.75)	0.002
Albumin, g/L	38.60 ± 3.80	35.60 ± 3.81	< 0.001
Prealbumin, mg/L	96.00 ± 25.00	78.01 ± 25.00	< 0.001
ALT, U/L	32.00 (18.00–58.00)	45.00 (25.00–82.00)	0.027
AST, U/L	34.00 (22.00–55.00)	48.00 (28.00–85.00)	0.042
Sodium, mmol/L	135.70 ± 2.70	133.90 ± 2.71	0.001
Ferritin, ng/mL	105.00 (55.00–180.10)	180.00 (90.00–320.00)	0.003
BNP, pg/mL	22.00 (8.00–54.80)	55.00 (18.00–140.00)	0.002
IL‐6, pg/mL	72.44 (26.39–190.66)	250.46 (200.00–350.50)	< 0.001
IL‐8, pg/mL	31.47 (16.77–66.51)	107.00 (87.90–149.50)	< 0.001
IL‐17A, pg/mL	5.96 (3.87–9.87)	33.80 (20.40–44.60)	< 0.001

*Note:* Data are mean ± SD or median (IQR). Conventional markers reflect systemic inflammation rather than coronary‐specific vascular injury.

Abbreviations: ALT, alanine aminotransferase; AST, aspartate aminotransferase; BNP, B‐type natriuretic peptide; CRP, C‐reactive protein; ESR, erythrocyte sedimentation rate; Hb, hemoglobin; HCT, hematocrit; IL, interleukin; PLT, platelet count; WBC, white blood cell count.

**Table 3:** Coronary artery measurements, recorded Z‐scores, and CAL severity.

Coronary measurement	Non‐CAL (*n* = 166)	CAL (*n* = 33)	*p* value
LMCA inner diameter, mm	2.50 ± 0.25	2.68 ± 0.44	0.028
LMCA Z‐score	0.30 (−0.18–0.71)	0.73 (−0.10–1.74)	0.004
LAD inner diameter, mm	1.94 ± 0.20	2.15 ± 0.44	0.009
LAD Z‐score	0.17 (−0.27–0.74)	0.93 (0.22–2.42)	< 0.001
RCA inner diameter, mm	1.89 ± 0.24	2.09 ± 0.40	0.008
RCA Z‐score	0.17 (−0.29–0.76)	0.75 (0.37–2.12)	< 0.001
LCX inner diameter, mm	1.75 ± 0.20	2.09 ± 1.09	0.083
LCX Z‐score	0.21 (−0.33–0.78)	0.74 (0.01–2.23)	0.001
Maximum Z‐score	1.04 (0.63–1.30)	2.62 (2.23–3.81)	< 0.001
Dilation only, *n* (%)	0 (0.0)	15 (45.5)	NA
Small aneurysm, *n* (%)	0 (0.0)	12 (36.4)	NA
Medium aneurysm, *n* (%)	0 (0.0)	5 (15.2)	NA
Large/giant aneurysm, *n* (%)	0 (0.0)	1 (3.0)	NA
Single‐segment involvement, *n* (%)	0 (0.0)	24 (72.7)	NA
Two‐segment involvement, *n* (%)	0 (0.0)	9 (27.3)	NA

*Note:* CAL was defined using the maximum segment‐level recorded Z‐score (≥ 2.0). Severity categories followed AHA criteria: dilation only, Z 2.0 to < 2.5; small aneurysm, Z 2.5 to < 5.0; medium aneurysm, Z 5.0 to < 10.0 with absolute dimension < 8 mm; and large/giant aneurysm, Z ≥ 10.0 or absolute dimension ≥ 8 mm. The archived database did not retain the Z‐score equation/software version. Individual CAL‐case measurements are provided in Table S1.

Abbreviations: LAD, left anterior descending; LCX, left circumflex; LMCA, left main coronary artery; RCA, right coronary artery.

### 3.4 Primary Association Model

**Figure 3:**
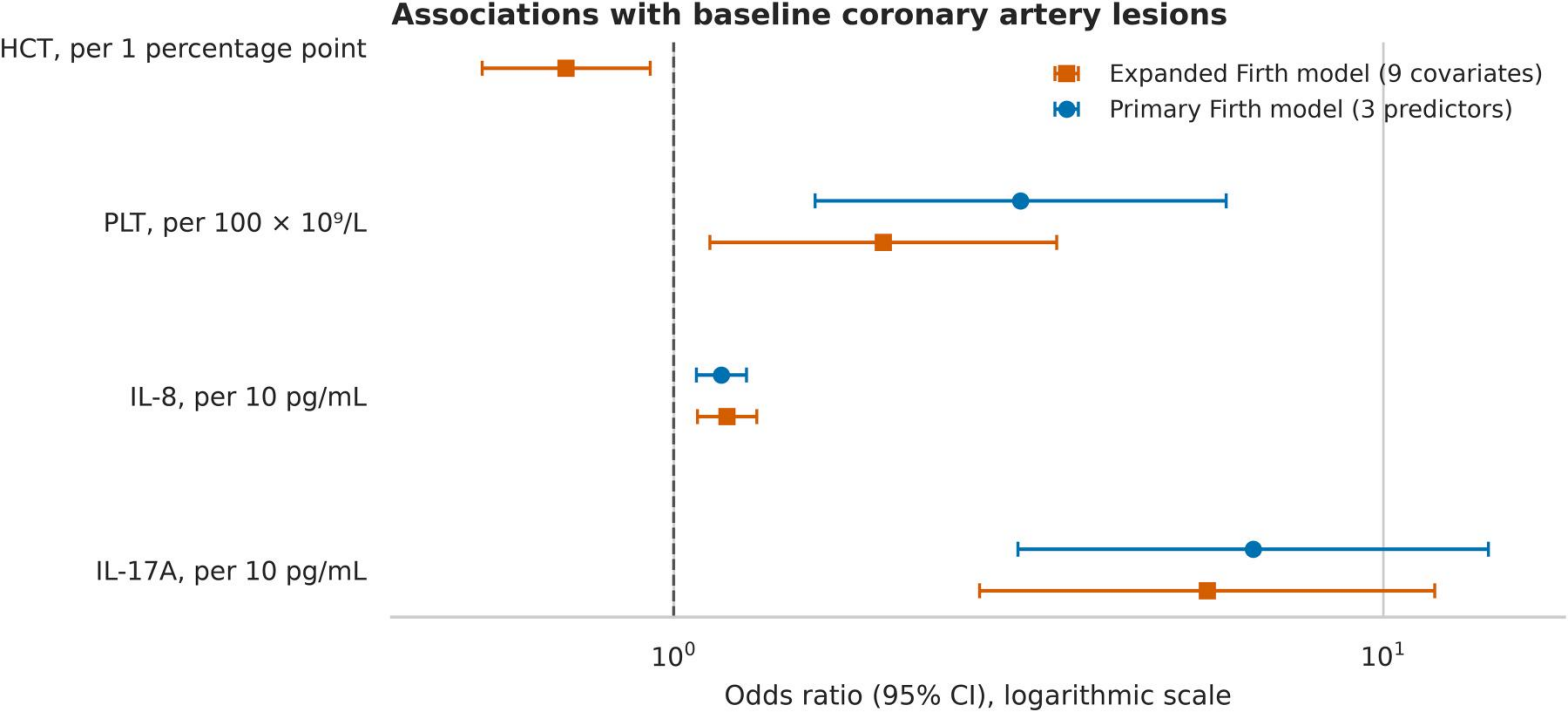
Firth‐penalized associations with baseline CAL in 199 children, including 33 with CAL. The reduced primary model contained PLT, IL‐8, and IL‐17A (11 events per predictor). The exploratory expanded model contained age, sex, illness day, albumin, sodium, HCT, PLT, IL‐8, and IL‐17A. Points denote ORs and horizontal lines denote 95% approximate Wald CIs. HCT is reported per 1 percentage point, PLT per 100 × 10^9^/L, and cytokines per 10 pg/mL. CAL, coronary artery lesion; CI, confidence interval; HCT, hematocrit; OR, odds ratio; PLT, platelet count.

With 33 CAL events and three predictors in the reduced primary model (11 events per predictor), the Firth model showed associations of baseline CAL with PLT (OR, 3.081 per 100 × 10^9^/L; 95% CI, 1.582–6.002; *p* < 0.001), IL‐8 (OR, 1.167 per 10 pg/mL; 95% CI, 1.076–1.266; *p* < 0.001), and IL‐17A (OR, 6.555 per 10 pg/mL; 95% CI, 3.054–14.071; *p* < 0.001) (Table 4 and Figure 3). The corresponding maximum‐likelihood estimates were directionally concordant (Table S3).

### 3.5 Expanded and Confounder Sensitivity Analyses

The exploratory nine‐covariate Firth model retained associations for lower HCT and higher PLT, IL‐8, and IL‐17A (Table 5 and Figure 3). In separate models that added admission illness day plus incomplete‐KD status or fever duration before IVIG plus incomplete‐KD status to the three primary biomarkers, the biomarker estimates remained directionally consistent, whereas the timing and incomplete‐KD covariates were not statistically significant (Table 6). IVIG resistance was not included because it occurred after baseline outcome ascertainment.

**Table 4:** Primary firth‐penalized logistic model for baseline CAL.

Variable	β	SE	OR (95% CI)	*p* value
PLT, per 100 × 10^9^/L	1.125	0.340	3.081 (1.582–6.002)	< 0.001
IL‐8, per 10 pg/mL	0.154	0.041	1.167 (1.076–1.266)	< 0.001
IL‐17A, per 10 pg/mL	1.880	0.390	6.555 (3.054–14.071)	< 0.001

*Note:* The reduced primary model contained PLT, IL‐8, and IL‐17A for 33 CAL events (11 events per predictor). Firth bias reduction was used. CI, approximate Wald confidence interval; OR, odds ratio.

**Table 5:** Exploratory expanded Firth‐penalized model.

Variable	β	SE	OR (95% CI)	*p* value
Age, per year	−0.290	0.169	0.749 (0.538–1.042)	0.086
Male sex	−0.676	0.795	0.508 (0.107–2.414)	0.395
Illness day, per day	0.048	0.204	1.049 (0.704–1.564)	0.814
Albumin, per g/L	−0.113	0.105	0.893 (0.726–1.098)	0.283
Sodium, per mmol/L	−0.186	0.136	0.830 (0.636–1.083)	0.170
HCT, per percentage point	−0.349	0.139	0.705 (0.537–0.926)	0.012
PLT, per 100 × 10^9^/L	0.680	0.287	1.974 (1.124–3.464)	0.018
IL‐8, per 10 pg/mL	0.173	0.049	1.189 (1.080–1.310)	< 0.001
IL‐17A, per 10 pg/mL	1.731	0.377	5.647 (2.699–11.811)	< 0.001

*Note:* This nine‐covariate model is exploratory because only 33 CAL events were available. Estimates are not a deployable clinical rule.

**Table 6:** Illness‐timing and incomplete‐KD Firth sensitivity analyses.

Model	Variable	OR	95% CI	*p* value
Admission‐day model	Illness day	1.351	0.888–2.057	0.160
Admission‐day model	Incomplete KD	2.331	0.511–10.633	0.275
Admission‐day model	PLT	3.011	1.517–5.977	0.002
Admission‐day model	IL‐8	1.167	1.075–1.267	< 0.001
Admission‐day model	IL‐17A	6.048	2.837–12.891	< 0.001
Fever‐duration model	Fever duration	1.402	0.916–2.146	0.120
Fever‐duration model	Incomplete KD	2.195	0.490–9.838	0.304
Fever‐duration model	PLT	2.984	1.504–5.923	0.002
Fever‐duration model	IL‐8	1.175	1.081–1.279	< 0.001
Fever‐duration model	IL‐17A	6.351	2.914–13.840	< 0.001

*Note:* Each model included the three primary biomarkers plus the stated timing variable and incomplete‐KD status. PLT is per 100 × 10^9^/L; cytokines are per 10 pg/mL. IVIG resistance was post‐outcome and not adjusted for.

### 3.6 Discrimination and Internal Validation

**Figure 4:**
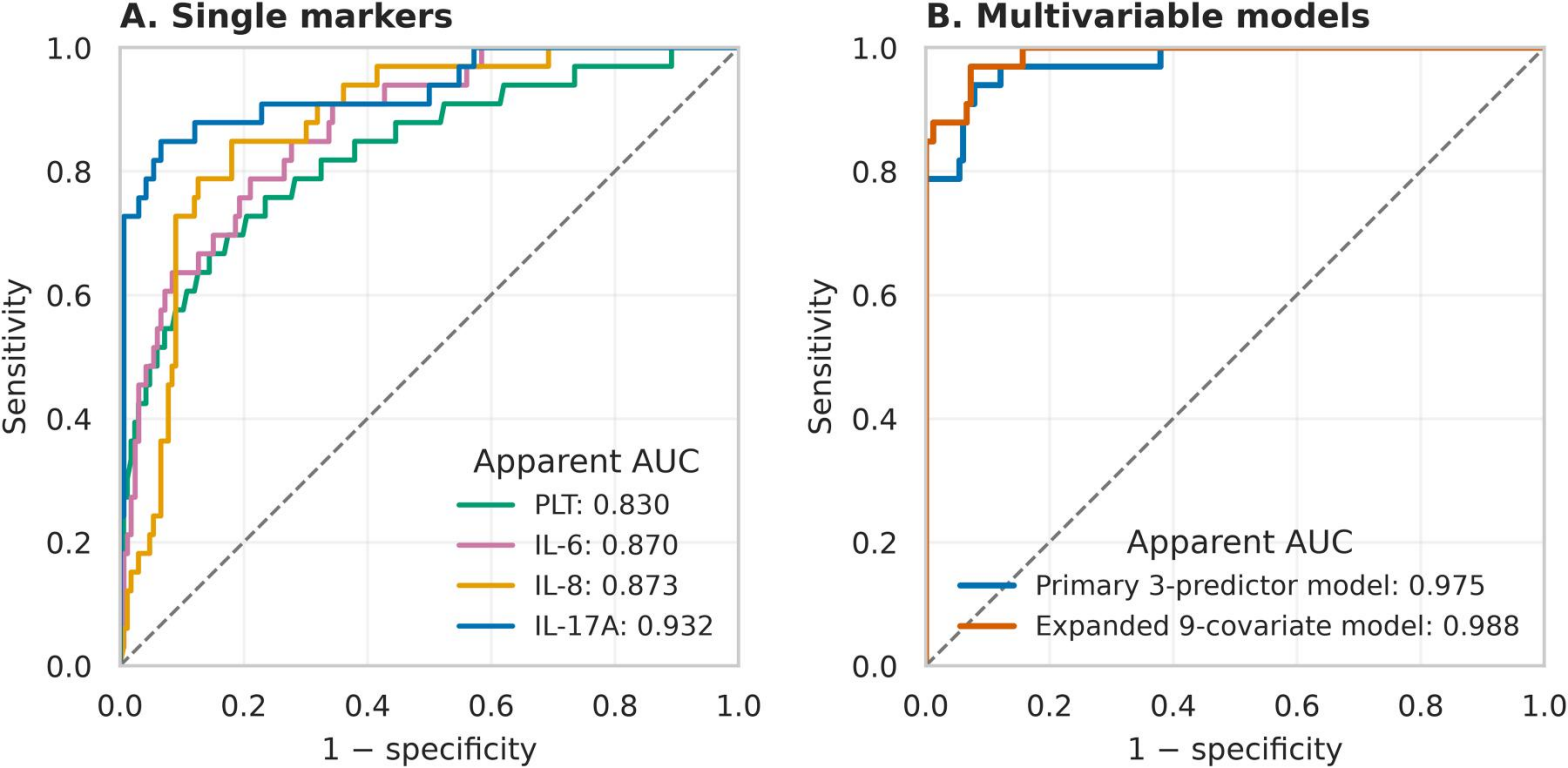
Apparent discrimination in the derivation cohort (*n* = 199; 33 with CAL). (A) Single‐marker receiver operating characteristic curves. (B) Multivariable curves for the primary three‐predictor and exploratory nine‐covariate models. Apparent AUCs should not be interpreted as external performance. Bootstrap and repeated‐cross‐validation results for the primary model are reported in Table 8. AUC, area under the curve; CAL, coronary artery lesion; IL, interleukin; PLT, platelet count.

IL‐17A had the highest apparent single‐marker AUC (0.932; 95% CI, 0.876–0.987), followed by IL‐8 (0.873), IL‐6 (0.870), and PLT (0.830) (Table 7 and Figure 4A). The primary three‐predictor model had an apparent AUC of 0.975. After 1000 bootstrap resamples, mean optimism was 0.003 and the optimism‐corrected AUC was 0.972. Across 100 repetitions of stratified fivefold cross‐validation, the mean AUC was 0.966 (SD, 0.006; empirical 95% interval, 0.951–0.972) (Table 8). The expanded model had higher apparent discrimination but less stable cross‐validated performance, consistent with overfitting risk (Figure 4B and Table 8).

**Table 7:** Apparent single‐marker discrimination.

Marker	AUC	95% CI	AUC difference versus IL‐17A	DeLong *p* value
PLT	0.830	0.746–0.914	0.101	0.049
IL‐6	0.870	0.808–0.932	0.061	0.151
IL‐8	0.873	0.814–0.932	0.059	0.175
IL‐17A	0.932	0.876–0.987	Reference	Reference

*Note:* AUCs are apparent derivation‐cohort estimates. DeLong tests were exploratory and unadjusted for multiplicity. AUC, area under the receiver operating characteristic curve.

**Table 8:** Internal validation and model‐complexity comparison.

Model	Apparent AUC	Bootstrap optimism	Optimism‐corrected AUC	Repeated‐CV AUC, mean ± SD (95% interval)
Primary Firth model (3 predictors)	0.975	0.003	0.972	0.966 ± 0.006 (0.951–0.972)
Expanded ML model (9 covariates)	0.988	0.020	0.969	0.933 ± 0.029 (exploratory)
Expanded Firth model (9 covariates)	0.989	Not repeated	Not estimated	Not estimated

*Note:* The primary model used 1000 successful bootstrap resamples and 100 repetitions of stratified fivefold cross‐validation with full refitting. The expanded maximum‐likelihood results are retained as exploratory context and show greater validation instability.

Abbreviations: AUC, area under the curve; CV, cross‐validation; ML, maximum likelihood; SD, standard deviation.

### 3.7 Additional Robustness Analyses

Low variance‐inflation factors argued against major linear collinearity. IL‐17A was not correlated with illness day in the full cohort or the overlapping illness‐day range, and associations remained directionally consistent in illness‐day‐restricted and log_2_ cytokine analyses. Complete 12‐cytokine results and false‐discovery‐rate‐adjusted comparisons are provided in Tables S2 and S3.

The complete set of expanded, nested‐model, CRP‐adjusted, log_2_‐scale, illness‐day‐restricted, and correlation analyses is reported in Table S3 so that the reduced primary analysis remains distinct from exploratory sensitivity analyses.

## 4 Discussion

This study found that a focused pre‐IVIG profile comprising IL‐17A, IL‐8, and PLT was associated with baseline Z‐score‐defined CAL. Restricting the primary model to three predictors provided 11 events per predictor, and Firth penalization further addressed small‐sample bias. The high apparent AUC changed little after bootstrap correction, although single‐center internal validation cannot establish transportability. The findings support replication and prospective validation, not a clinical prediction rule or causal inference.

The IL‐17A association is biologically compatible with prior work but is not mechanistic evidence from this cohort. Existing proteomic and experimental studies have linked IL‐17‐family signaling with endothelial activation, IL‐8 expression, neutrophil recruitment, and coronary arteritis [27, 28, 29, 30, 31]. We measured circulating cytokines once and performed no cell, tissue, animal, or intervention experiment. Thus, the graphical and textual pathway discussion summarizes prior literature only; it must not be read as demonstrating mediation or causality in these patients.

The concurrent IL‐17A and IL‐8 signals largely replicate and extend prior clinical observations [23, 24, 27, 28, 29]. Their simultaneous retention in adjusted models is compatible with, but does not prove, linked inflammatory biology. The principal incremental contribution here is methodological and phenotypic: pre‐IVIG measurement, segment‐level recorded Z‐score outcome definition, reduced‐complexity Firth modeling, and explicit bootstrap/cross‐validation. Novel biomarkers and experimental pathways were outside the study design.

IL‐6 deserves a cautious interpretation. It was higher in the CAL group in univariate analysis and showed substantial apparent single‐marker discrimination, consistent with its role as a dynamic marker of systemic inflammation [25, 26]. In this cohort, children with CAL were sampled later in the illness course and also showed broader inflammatory and biochemical differences, so the univariate IL‐6 separation may reflect inflammatory burden, illness timing, or both. With only 33 CAL events, adding IL‐6 to the reduced primary model would increase predictor burden and undermine the model‐complexity restriction adopted in response to the small event count. IL‐6 was therefore retained as a transparent univariate and single‐marker finding rather than used to support an independent multivariable association. This modeling decision should not be interpreted as evidence that IL‐6 is biologically irrelevant.

Higher PLT was associated with CAL in both reduced and expanded analyses, whereas lower HCT appeared only in the expanded model. These patterns are consistent with known hematologic changes in KD but remain observational. Reporting HCT per 1 percentage point and PLT per 100 × 10^9^/L provides clinically interpretable effect scales.

The contribution of this study lies in integrated clinical phenotyping and analytic evaluation rather than biomarker discovery. In a pre‐IVIG cohort, we contextualized IL‐17A and IL‐8 within the complete measured cytokine panel, evaluated these signals alongside PLT against recorded segment‐level Z‐score‐defined coronary involvement, and used reduced‐complexity Firth modeling with repeated internal validation to address the limited number of CAL events. These findings should therefore be interpreted as independent clinical replication and hypothesis refinement rather than evidence of a novel mechanistic pathway or a deployable prediction tool.

Clinical implications should be concrete but conservative. These biomarkers should not independently alter echocardiographic frequency, adjunctive treatment, or follow‐up. Children with uncomplicated KD should continue to receive echocardiography at diagnosis and at guideline‐recommended follow‐up intervals (commonly approximately 1–2 weeks and 4–6 weeks). Earlier or more frequent imaging, cardiology escalation, and adjunctive anti‐inflammatory treatment should be based on established indications—including abnormal coronary Z‐scores, persistent or recurrent fever, IVIG resistance, or other guideline‐defined high‐risk features—rather than cytokine concentrations [1, 2, 10]. A biomarker‐guided schedule or treatment threshold would require prospective external validation and clinical‐utility analysis.

Strengths include recovery of segment‐level recorded coronary measurements, a reduced primary model, Firth bias reduction, and repeated internal validation. Limitations remain substantial. First, the retrospective single‐center design and 33 events limit precision; the nine‐covariate model remains overfit‐prone and is explicitly exploratory. Second, residual confounding by illness phase, incomplete phenotype, referral patterns, or unmeasured severity cannot be excluded despite timing and incomplete‐KD sensitivity analyses. Third, although recorded segmental Z‐scores were recovered, the archived database did not retain the equation or software version, reducing reproducibility and cross‐study comparability. Fourth, complete‐case and cytokine‐availability selection may have introduced bias. Fifth, single pre‐IVIG measurements preclude trajectory analysis. Sixth, few medium or giant aneurysms prevented severity‐specific modeling. Finally, observational associations cannot establish mechanism, mediation, clinical utility, or causality.

Future multicenter studies should prospectively prespecify the coronary Z‐score equation, sampling window, reduced predictor set, and external‐validation plan; repeat cytokine measurements; and evaluate calibration, decision curves, and clinical consequences before any biomarker‐guided care is considered.

## 5 Conclusion

In this retrospective exploratory cohort, higher pre‐IVIG IL‐17A, IL‐8, and PLT were associated with baseline Z‐score‐defined CAL. A three‐predictor Firth model reduced, but did not eliminate, small‐sample overfitting concerns and showed high internally validated discrimination. IL‐6 showed a marked univariate difference but was not entered into the reduced multivariable model because of event‐count constraints; no inference regarding an independent IL‐6 effect is made. These data replicate a clinical inflammatory signal without establishing mechanism or supporting changes to imaging or treatment. Prospective multicenter external validation is required.

## Author Contributions

Cuijie Gong and Yahui Lin contributed equally to this work. Cuijie Gong and Pan Li conceived and designed the study. Cuijie Gong, Yahui Lin, Yanmei Lang, Xinfeng Liu, Dandan Li, and Sha Wang collected and organized the clinical, laboratory, cytokine, and echocardiographic data. Cuijie Gong and Yahui Lin performed the statistical analysis and interpreted the results. Cuijie Gong drafted the initial manuscript. Yahui Lin, Yanmei Lang, Xinfeng Liu, Dandan Li, and Sha Wang critically revised the manuscript for important intellectual content. Pan Li supervised the study, verified the data interpretation, and revised the manuscript. All authors read and approved the final manuscript.

## Ethics Statement

This retrospective study involving human clinical data was approved by the Ethics Committee of Hebei Children's Hospital (approval No. 202136). The study was performed in accordance with the Declaration of Helsinki, and written informed consent for the use of clinical data was obtained from the legal guardians of all participants according to local institutional requirements.

## Conflicts of Interest

The authors declare no conflicts of interest.

## Supporting information


Supporting File 1

Supporting File 2

Supporting File 3

Supporting File 4

## Data Availability

The de‐identified datasets used and/or analysed during the current study are available from the corresponding author on reasonable request, subject to institutional data‐sharing regulations and applicable privacy requirements.
