# Supplementary material for: Interleukin‐17A, Interleukin‐8, and Platelet Count Are Associated With Baseline Coronary Artery Lesions in Children With Kawasaki Disease: A Retrospective Exploratory Cohort Study
Source: Immun Inflamm Dis. 2026 Sep 24;14(9):e70514. doi: 10.1002/iid3.70514 (PMC13601989; doi:10.1002/iid3.70514)
Supplement: Supplementary file 2 [file IID3-14-e70514-s002.docx]

**Supplementary Table S3. Robustness and secondary analyses**

**Supplementary Table S3A. Primary conventional and Firth estimates**

| **Estimator** | **Variable** | **OR** | **95% CI** | **P value** |
| --- | --- | --- | --- | --- |
| Maximum likelihood | PLT | 3.538 | 1.620–7.725 | 0.002 |
| Maximum likelihood | IL-8 | 1.182 | 1.077–1.297 | <0.001 |
| Maximum likelihood | IL-17A | 8.295 | 3.272–21.034 | <0.001 |
| Firth | PLT | 3.081 | 1.582–6.002 | <0.001 |
| Firth | IL-8 | 1.167 | 1.076–1.266 | <0.001 |
| Firth | IL-17A | 6.555 | 3.054–14.071 | <0.001 |

*PLT is reported per 100 × 10⁹/L; cytokines are reported per 10 pg/mL.*

**Supplementary Table S3B. Exploratory nested-model internal validation**

| **Model** | **Apparent AUC** | **Optimism-corrected AUC** | **Repeated-CV AUC, mean ± SD** | **Paired CV ΔAUC vs clinical model (95% interval)** |
| --- | --- | --- | --- | --- |
| Clinical model | 0.922 | 0.895 | 0.881 ± 0.011 | Reference |
| Clinical + IL-8 | 0.943 | 0.917 | 0.900 ± 0.012 | 0.020 (0.004–0.034) |
| Clinical + IL-17A | 0.978 | 0.958 | 0.936 ± 0.018 | 0.055 (0.018–0.079) |
| Full model | 0.988 | 0.969 | 0.933 ± 0.029 | 0.052 (−0.0004–0.094) |

*The clinical model included age, sex, illness day, albumin, sodium, HCT, and PLT. All nested-model analyses are exploratory.*

**Supplementary Table S3C. CRP-base-model sensitivity analysis**

| **Model** | **Ordinary apparent AUC** | **Firth apparent AUC** | **Repeated-CV AUC, mean ± SD** | **Firth IL-17A OR (95% CI)** |
| --- | --- | --- | --- | --- |
| Clinical + CRP | 0.935 | 0.934 | 0.892 ± 0.010 | — |
| Clinical + CRP + IL-17A | 0.980 | 0.980 | 0.939 ± 0.021 | 5.703 (2.518–12.916) |

*The paired mean CV AUC difference after adding IL-17A was 0.047 (95% empirical interval, −0.002 to 0.069).*

**Supplementary Table S3D. Log₂-scale and illness-day sensitivity analyses**

| **Analysis** | **Variable** | **Estimate** | **P value** | **Additional information** |
| --- | --- | --- | --- | --- |
| Log₂ model | IL-8, per two-fold increase | 3.485 (1.546–7.855) | 0.003 | n = 199 |
| Log₂ model | IL-17A, per two-fold increase | 6.201 (2.404–15.992) | <0.001 | n = 199 |
| Illness-day overlap restriction | IL-17A, per 10 pg/mL | 5.536 (2.505–12.233) | <0.001 | Days 4–9; n = 181; apparent AUC = 0.932 |
| Illness-day correlation | IL-17A vs illness day | ρ = 0.067 | 0.344 | Full cohort |
| Illness-day correlation | IL-17A vs illness day | ρ = 0.082 | 0.271 | Days 4–9 |

*Log₂-model ORs represent a two-fold increase in cytokine concentration.*

**Supplementary Table S3E. Variance-inflation-factor diagnostics**

| **Variable** | **VIF** | **Interpretation** |
| --- | --- | --- |
| Age | 1.027 | Low |
| Male sex | 1.059 | Low |
| Illness day | 1.046 | Low |
| Albumin | 1.112 | Low |
| Sodium | 1.089 | Low |
| HCT | 1.118 | Low |
| PLT | 1.226 | Low |
| IL-8 | 1.546 | Low |
| IL-17A | 1.352 | Low |

*All VIF values were below 2.*
