# Supplementary material for: Interleukin‐17A, Interleukin‐8, and Platelet Count Are Associated With Baseline Coronary Artery Lesions in Children With Kawasaki Disease: A Retrospective Exploratory Cohort Study
Source: Immun Inflamm Dis. 2026 Sep 24;14(9):e70514. doi: 10.1002/iid3.70514 (PMC13601989; doi:10.1002/iid3.70514)
Supplement: Supplementary file 3 [file IID3-14-e70514-s003.docx]

**Supplementary Table S1A. Individual characteristics and CAL classification for all 33 CAL cases**

| **Patient ID** | **Age, y** | **Sex** | **Height, cm** | **Weight, kg** | **BSA, m²** | **Maximum Z** | **Involved segment(s)** | **CAL severity** |
| --- | --- | --- | --- | --- | --- | --- | --- | --- |
| KD2023-166 | 3.42 | Female | 91.4 | 12.1 | 0.554 | 7.51 | LAD, RCA | Medium aneurysm |
| KD2023-167 | 0.17 | Male | 52.7 | 4.3 | 0.251 | 7.58 | LMCA | Medium aneurysm |
| KD2023-168 | 1.67 | Male | 86.2 | 12.2 | 0.540 | 3.81 | LAD | Small aneurysm |
| KD2023-169 | 2.08 | Male | 85.7 | 11.7 | 0.528 | 2.43 | LCX | Dilation only |
| KD2023-170 | 3.25 | Male | 93.0 | 15.5 | 0.633 | 2.20 | RCA | Dilation only |
| KD2023-171 | 4.92 | Male | 108.1 | 21.4 | 0.802 | 2.42 | LAD | Dilation only |
| KD2023-172 | 0.17 | Male | 53.0 | 4.5 | 0.257 | 5.31 | LAD, RCA | Medium aneurysm |
| KD2023-173 | 3.67 | Female | 101.6 | 18.0 | 0.713 | 2.36 | RCA | Dilation only |
| KD2023-174 | 0.17 | Male | 52.0 | 4.3 | 0.249 | 2.86 | LAD, LCX | Small aneurysm |
| KD2023-175 | 0.17 | Male | 52.0 | 4.8 | 0.263 | 10.97 | LCX | Large/giant aneurysm |
| KD2023-176 | 4.58 | Female | 103.4 | 17.3 | 0.705 | 2.66 | LMCA, RCA | Small aneurysm |
| KD2023-177 | 4.00 | Male | 98.8 | 14.0 | 0.620 | 3.80 | LAD | Small aneurysm |
| KD2023-178 | 1.92 | Male | 93.4 | 12.4 | 0.567 | 2.62 | LAD | Small aneurysm |
| KD2023-179 | 0.83 | Male | 72.3 | 9.7 | 0.441 | 2.27 | LAD | Dilation only |
| KD2023-180 | 0.17 | Female | 52.0 | 4.1 | 0.243 | 4.37 | RCA | Small aneurysm |
| KD2023-181 | 5.33 | Female | 106.9 | 21.4 | 0.797 | 5.33 | LMCA, LCX | Medium aneurysm |
| KD2023-182 | 0.17 | Male | 52.0 | 4.7 | 0.261 | 3.14 | LMCA, LCX | Small aneurysm |
| KD2023-183 | 0.58 | Male | 60.6 | 5.9 | 0.315 | 2.16 | LCX | Dilation only |
| KD2023-184 | 2.50 | Male | 92.4 | 14.8 | 0.616 | 2.21 | LAD | Dilation only |
| KD2023-185 | 5.92 | Female | 113.7 | 23.7 | 0.865 | 2.03 | RCA | Dilation only |
| KD2023-186 | 4.25 | Male | 100.3 | 17.1 | 0.690 | 2.06 | LCX | Dilation only |
| KD2023-187 | 0.17 | Male | 52.0 | 4.1 | 0.243 | 2.63 | LAD, RCA | Small aneurysm |
| KD2023-188 | 0.17 | Female | 53.2 | 4.3 | 0.252 | 2.68 | LCX | Small aneurysm |
| KD2023-189 | 2.33 | Male | 86.0 | 13.5 | 0.568 | 4.42 | LMCA, LCX | Small aneurysm |
| KD2023-190 | 6.58 | Male | 116.6 | 21.6 | 0.836 | 3.51 | LCX | Small aneurysm |
| KD2023-191 | 1.08 | Male | 67.7 | 7.5 | 0.376 | 2.31 | RCA | Dilation only |
| KD2023-192 | 3.00 | Male | 89.9 | 12.7 | 0.563 | 2.23 | LCX | Dilation only |
| KD2023-193 | 1.50 | Male | 81.3 | 12.3 | 0.527 | 2.12 | RCA | Dilation only |
| KD2023-194 | 0.33 | Female | 57.6 | 5.3 | 0.291 | 2.60 | LMCA, LAD | Small aneurysm |
| KD2023-195 | 1.25 | Female | 70.5 | 7.0 | 0.370 | 7.75 | LAD | Medium aneurysm |
| KD2023-196 | 0.17 | Male | 52.0 | 3.8 | 0.234 | 2.27 | LMCA | Dilation only |
| KD2023-198 | 2.75 | Male | 91.8 | 14.4 | 0.606 | 2.04 | RCA | Dilation only |
| KD2023-199 | 7.92 | Male | 121.9 | 23.6 | 0.894 | 2.10 | LAD | Dilation only |

Patient IDs are de-identified study codes. S1A and S1B use the identical patient order. BSA was calculated using the Mosteller formula for descriptive reporting and was not used to recompute the archived clinical Z-scores. CAL severity followed AHA criteria: dilation only, Z 2.0 to <2.5; small aneurysm, Z 2.5 to <5.0; medium aneurysm, Z 5.0 to <10.0 with absolute dimension <8 mm; and large/giant aneurysm, Z ≥10.0 or absolute dimension ≥8 mm. BSA, body surface area; CAL, coronary artery lesion.

**Supplementary Table S1B. Absolute coronary diameters and segmental Z-scores for all 33 CAL cases**

| **Patient ID** | **LMCA, mm** | **LMCA Z** | **LAD, mm** | **LAD Z** | **RCA, mm** | **RCA Z** | **LCX, mm** | **LCX Z** |
| --- | --- | --- | --- | --- | --- | --- | --- | --- |
| KD2023-166 | 2.58 | 0.32 | 3.27 | 6.35 | 3.58 | 7.51 | 1.90 | 0.71 |
| KD2023-167 | 3.88 | 7.58 | 1.86 | 1.11 | 1.71 | 0.56 | 1.44 | -0.12 |
| KD2023-168 | 2.35 | -0.40 | 2.74 | 3.81 | 2.01 | 0.68 | 1.72 | 0.01 |
| KD2023-169 | 2.88 | 1.74 | 2.08 | 1.06 | 1.93 | 0.50 | 2.27 | 2.43 |
| KD2023-170 | 2.42 | -0.29 | 1.92 | 0.07 | 2.44 | 2.20 | 1.78 | 0.07 |
| KD2023-171 | 2.86 | 0.66 | 2.64 | 2.42 | 2.02 | 0.03 | 1.68 | -1.15 |
| KD2023-172 | 2.34 | 0.73 | 2.24 | 3.02 | 2.53 | 5.31 | 1.62 | 0.74 |
| KD2023-173 | 2.62 | -0.09 | 2.20 | 0.57 | 2.53 | 2.36 | 2.00 | 0.73 |
| KD2023-174 | 2.02 | -0.22 | 2.12 | 2.61 | 1.91 | 1.80 | 2.01 | 2.86 |
| KD2023-175 | 2.25 | 0.76 | 1.81 | 0.74 | 1.74 | 0.75 | 7.80 | 10.97 |
| KD2023-176 | 3.29 | 2.66 | 1.76 | -0.95 | 2.46 | 2.17 | 2.11 | 1.44 |
| KD2023-177 | 2.39 | -0.59 | 2.84 | 3.80 | 1.75 | -0.41 | 1.78 | 0.11 |
| KD2023-178 | 2.58 | 0.45 | 2.46 | 2.62 | 1.67 | -0.86 | 1.91 | 0.80 |
| KD2023-179 | 2.27 | -0.38 | 2.35 | 2.27 | 2.05 | 1.26 | 1.66 | -0.15 |
| KD2023-180 | 2.38 | 1.29 | 1.58 | 0.10 | 2.44 | 4.37 | 1.51 | 0.20 |
| KD2023-181 | 3.69 | 3.64 | 1.88 | -0.73 | 2.38 | 1.30 | 3.06 | 5.33 |
| KD2023-182 | 2.85 | 3.14 | 1.84 | 0.93 | 1.61 | -0.06 | 1.94 | 2.43 |
| KD2023-183 | 2.17 | -0.28 | 1.58 | -0.65 | 1.71 | 0.42 | 1.94 | 2.16 |
| KD2023-184 | 2.69 | 0.83 | 2.42 | 2.21 | 1.86 | -0.13 | 1.67 | -0.51 |
| KD2023-185 | 2.74 | -0.10 | 2.20 | 0.27 | 2.54 | 2.03 | 1.97 | 0.00 |
| KD2023-186 | 2.75 | 0.65 | 2.03 | 0.22 | 2.01 | 0.30 | 2.27 | 2.06 |
| KD2023-187 | 2.29 | 0.73 | 2.05 | 2.43 | 2.08 | 2.63 | 1.79 | 1.33 |
| KD2023-188 | 2.30 | 0.72 | 1.92 | 1.34 | 1.71 | 0.51 | 1.95 | 2.68 |
| KD2023-189 | 3.62 | 4.42 | 1.72 | -0.94 | 2.05 | 0.93 | 2.58 | 4.12 |
| KD2023-190 | 2.91 | 0.78 | 2.25 | 0.71 | 2.07 | 0.20 | 2.69 | 3.51 |
| KD2023-191 | 2.58 | 1.19 | 1.79 | 0.17 | 2.15 | 2.31 | 1.53 | -0.46 |
| KD2023-192 | 2.93 | 1.80 | 1.94 | 0.25 | 2.00 | 0.37 | 2.19 | 2.23 |
| KD2023-193 | 2.31 | -0.50 | 1.99 | 0.72 | 2.34 | 2.12 | 1.87 | 0.79 |
| KD2023-194 | 2.84 | 2.60 | 2.12 | 2.21 | 1.75 | 0.61 | 1.59 | 0.30 |
| KD2023-195 | 2.46 | 0.57 | 3.34 | 7.75 | 1.89 | 1.03 | 1.60 | 0.13 |
| KD2023-196 | 2.56 | 2.27 | 1.53 | -0.47 | 1.60 | 0.56 | 1.43 | -0.19 |
| KD2023-198 | 2.47 | -0.18 | 1.98 | 0.38 | 2.36 | 2.04 | 2.09 | 1.60 |
| KD2023-199 | 3.13 | 1.44 | 2.63 | 2.10 | 2.05 | -0.11 | 1.72 | -0.96 |

Patient IDs are de-identified study codes. LAD, left anterior descending artery; LCX, left circumflex artery; LMCA, left main coronary artery; RCA, right coronary artery.
