# Supplementary material for: Interleukin‐17A, Interleukin‐8, and Platelet Count Are Associated With Baseline Coronary Artery Lesions in Children With Kawasaki Disease: A Retrospective Exploratory Cohort Study
Source: Immun Inflamm Dis. 2026 Sep 24;14(9):e70514. doi: 10.1002/iid3.70514 (PMC13601989; doi:10.1002/iid3.70514)
Supplement: Supplementary file 4 [file IID3-14-e70514-s004.docx]

**Supplementary Table S2. Complete measured cytokine panel by baseline CAL status**

| **Cytokine, pg/mL** | **Non-CAL (n = 166), median (IQR)** | **CAL (n = 33), median (IQR)** | ***P* value** | **BH-adjusted q value** |
| --- | --- | --- | --- | --- |
| IL-2 | 2.00 (1.33–3.03) | 2.56 (1.43–3.68) | 0.126 | 0.189 |
| IL-4 | 3.01 (2.19–4.07) | 3.12 (2.18–4.57) | 0.294 | 0.353 |
| IL-6 | 72.44 (26.39–190.66) | 250.46 (200.00–350.50) | <0.001 | <0.001 |
| IL-8 | 31.47 (16.77–66.51) | 107.00 (87.90–149.50) | <0.001 | <0.001 |
| IL-10 | 11.98 (8.25–18.25) | 17.67 (10.14–30.36) | 0.006 | 0.017 |
| TNF-α | 4.33 (2.94–6.56) | 6.71 (3.92–8.72) | 0.011 | 0.027 |
| IFN-γ | 5.87 (3.83–8.97) | 5.69 (3.52–7.99) | 0.992 | 0.992 |
| IL-17A | 5.96 (3.87–9.87) | 33.80 (20.40–44.60) | <0.001 | <0.001 |
| IL-1β | 3.94 (2.55–5.48) | 5.23 (2.62–7.89) | 0.043 | 0.073 |
| IL-5 | 3.13 (2.35–4.32) | 3.38 (2.16–4.60) | 0.836 | 0.912 |
| IL-12p70 | 3.13 (2.19–4.64) | 3.78 (2.70–5.30) | 0.025 | 0.049 |
| IFN-α | 6.41 (4.05–8.71) | 7.07 (4.81–10.49) | 0.225 | 0.299 |

P values are two-sided Mann–Whitney comparisons. The Benjamini–Hochberg procedure controlled the false discovery rate across all 12 measured cytokines. BH, Benjamini–Hochberg; CAL, coronary artery lesion; IQR, interquartile range.
